# Erratum

**DOI:** 10.4269/ajtmh.17-0648err

**Published:** 2018-09

In *Am J Trop Med Hyg 99 (1):* 171–179 (https://doi.org/10.4269/ajtmh.17-0648) Saadiyah Bilal and Eric Nelson should have been denoted as co-first authors on the manuscript. In addition, the following line should have been included in the financial support section of the manuscript: “Software development was supported by the National Institutes of Health [DP5OD019893] to EJN.”

